# Involvement of the mitogen activated protein kinase Hog1p in the response of *Candida albicans* to iron availability

**DOI:** 10.1186/1471-2180-13-16

**Published:** 2013-01-24

**Authors:** Hani EJ Kaba, Manfred Nimtz, Peter P Müller, Ursula Bilitewski

**Affiliations:** 1 Working Group Biological Systems Analysis, Helmholtz Centre for Infection Research, Inhoffenstr. 7, D-38124 Braunschweig, Germany; 2 Working Group Cellular Proteomics, Helmholtz Centre for Infection Research, Inhoffenstr. 7, D-38124 Braunschweig, Germany; 3 Department Gene Regulation and Differentiation, Helmholtz Centre for Infection Research, Inhoffenstr. 7, D-38124 Braunschweig, Germany

**Keywords:** *Candida albicans*, HOG pathway, Iron, Flocculation, Multicopper ferroxidases, Ferric reductases

## Abstract

**Background:**

Iron is an essential nutrient for almost all organisms, and generating iron limiting conditions for pathogens is one of the host defense strategies against microbial infections. Excess of iron can be toxic; therefore, iron uptake is tightly controlled. The high affinity iron uptake system of the opportunistic pathogenic yeast *Candida albicans* has been shown to be essential for virulence. Several transcription factors and regulators of iron uptake genes were identified, but the knowledge of signaling pathways is still limited. Gene expression profiling of the Δ*hog1* deletion mutant indicated an involvement of the mitogen activated protein (MAP) kinase Hog1p. However, the function of Hog1p in the response of *C. albicans* to iron availability was not studied in detail. Thus, we analyzed phenotypic and molecular responses of *C. albicans* to different iron concentrations particularly with respect to the activity of the Hog1p MAP kinase module.

**Results:**

We observed flocculation of yeast cells, when the iron ion concentration was equal to or higher than 5 μM. This phenotype was dependent on the MAP kinase Hog1p and the corresponding MAP kinase kinase Pbs2p. Moreover, high extracellular iron ion concentrations led to hyper-phosphorylation of Hog1p. We determined lower amounts of multicopper ferroxidase (MCFO) proteins and lower ferric reductase activity, when the iron ion concentration in the medium was increased. This effect was also observed for the Δ*hog1* mutant. However, the amounts of MCFO proteins and the cell surface ferric reductase activity were increased in the Δ*hog1* in comparison to wild type cells. This effect was independent of iron availability in growth media.

**Conclusions:**

In *C. albicans*, the MAP kinase Hog1p is part of the network regulating the response of the organism to iron availability. Hog1p was transiently phosphorylated under high iron concentrations and was essential for a flocculent phenotype. Furthermore, deletion of *HOG1* led to increased levels of components of the reductive iron uptake system in comparison to the wild-type, independent of iron concentrations in the media. However, the additional induction of this system by low iron concentrations was independent of *HOG1.*

## Background

*Candida albicans* is an opportunistic fungal pathogen of humans and colonizes as commensal up to 30 – 70% of healthy individuals [1]. However, patients with a compromised immune system are at high risk to acquire systemic infections by *Candida spp.*, which constitute the fourth highest cause for nosocomial bloodstream infections with a lethality rate of up to 40% [2]. One of the reasons for the success of *C. albicans* as a pathogen is its high adaptability to various environmental niches, which are characterized by the availability of nutrients and essential elements.

Iron is essential for almost all organisms as it is a co-factor for a variety of proteins. It was shown that iron acquisition by pathogens is a limiting factor for fungal, bacterial and protozoan infections [3-5]. Pretreatment with iron chelators protected endothelial and epithelial cells from *C. albicans* mediated injury, while loading cells with iron reversed this effect [6,7]. Genes of iron acquisition proteins were upregulated during *C. albicans* liver tissue infection [8]. Moreover, iron availability was linked to drug resistance as well as to morphology of this fungus [9,10].

Iron accessibility for pathogens is restricted in mammalian hosts by proteins which bind iron with high affinity, such as hemoglobin, transferrin and ferritin. Pathogens have developed different strategies for iron acquisition to counteract this restricted iron environment inside the host.

Three systems for iron uptake by *C. albicans* are known: (i) A heme uptake system allowing the utilization of iron bound to hemoglobin, including hemoglobin receptors, e.g. Rbt5p [11,12]. (ii) The receptor Sit1p, which allows *C. albicans* to acquire iron from ferrichrome type siderophores [13,14]. Considering the lack of genes required for siderophore biosynthesis in *C. albicans*, it is believed that this pathway allows the uptake of iron bound to siderophores produced by other pathogens or commensals [15]. (iii) The reductive pathway, whereby ferric iron is reduced to ferrous iron by membrane associated ferric reductases [16], before it is reoxidized by members of the multicopper ferroxidase (MCFO) family [17]. MCFOs form together with the iron permease Ftr1p a high affinity iron uptake (HAIU) complex in the plasma membrane [18,19]. This pathway was shown to be responsible for iron uptake not only from iron salts but also from iron loaded host proteins such as transferrin and ferritin [7,20]. Deletion of *FTR1* rendered *C. albicans* completely avirulent in a mouse model and abolished the damage of oral epithelial cells [7,18]. Reduction of ferric iron to ferrous iron by reductases increases the solubility and availability of iron. However, the function of MCFOs leading to the reoxidation of Fe^2+^ is not as well understood. Complex formation with the permease and channeling of Fe^3+^ could maintain the availability of iron and deliver iron in the oxidized and less reactive form to the cytosol.

Due to the toxic potential of iron by generating reactive oxygen species (ROS) [21], cellular iron homeostasis is subjected to tight regulation. In *C. albicans*, the transcriptional regulators Sfu1p, Hap43p and Sef1p are part of an iron responsive regulatory network [22]. Sfu1p is a GATA-type repressor, which is active under high iron conditions. It negatively regulates genes encoding for ferric reductases, MCFOs, iron permeases, as well as Hap43p, the regulatory element of the CCAAT-binding complex (CBC) [22,23]. Hap43p is a transcription factor that is activated under low iron conditions and represses the expression of Sfu1p and of iron utilization genes so that repression of genes involved in iron uptake is relieved and the limited amount of iron is efficiently used for vital proteins [24]. Sef1p was identified as a transcriptional activator of iron uptake genes [25]. It is repressed by Sfu1p, but activated under low iron conditions. It induces Hap43p and iron uptake genes, such as *FET3* (encoding an MCFO), as well as a copper-transporting ATPase encoding gene (*CCC2*) required for MCFO activity [22]. Additionally, other transcription factors, such as Tup1p and Rim101p, are involved in the regulation of iron uptake genes, but their roles are not as obvious. Tup1p is a global repressor which may be recruited to iron responsive genes via interaction with Sfu1p [23], while regulation by Rim101p is influenced by pH [26].

This complex regulation of iron uptake probably helps *C. albicans* to successfully adapt to niches with different iron levels [22]. However, even though transcriptional regulators of the iron response network were identified, signaling pathways, which govern the activity of these regulators, are less well known.

Four iron uptake genes, namely the ferric reductase *FRE10*, the hemoglobin receptor *RBT5*, the high affinity iron permease *FTR1* and the MCFO *FET34*, were found to be de-repressed in cells lacking *HOG1* under sufficient iron conditions, which are usually repressive for these genes [27]. Hog1p encodes the mitogen activated protein kinase (MAPK) orthologous to human p38 [28] and to stress – activated protein kinases (SAPK) in other yeasts [27]. In response to several environmental stresses, Hog1p becomes phosphorylated and translocates to the nucleus [29]. *hog1* null mutants were found to be hypersensitive to those stress conditions, which lead to Hog1p activation, in particular to extracellular oxidizing agents [29,30]. At least the response to oxidative and osmotic stress depends on the mitogen activated protein kinase kinase Pbs2p [31]. Among the substrates of Hog1p are transcription factors [32] so that activation of Hog1p also modulates gene expression profiles [27].

As until now no further details are known on the regulatory role of Hog1p in the response of *C. albicans* to iron availability, we investigated phenotypic and molecular responses of *C. albicans* to extracellular iron levels. We observed flocculation of wild type (WT) cells with increasing iron concentrations. This phenotype was dependent on both protein synthesis and an intact HOG pathway as it was abolished in the Δ*hog1* and the Δ*pbs2* mutants. Moreover, deletion of *HOG1* led to the de-repression of MCFOs as wells as to increased ferric reductase activity under sufficient iron conditions. However, cultivation of the Δ*hog1* mutant in restricted iron medium enhanced the expression even further. Reactive oxygen species (ROS) were accumulated under excessive iron conditions in the WT as well as in the Δ*hog1* mutant thus indicating iron uptake by both strains. Moreover, in the WT we observed transient phosphorylation of Hog1p under high iron conditions.

## Results

### Iron induced *C. albicans* flocculation in a concentration dependent manner

**Figure 1:**
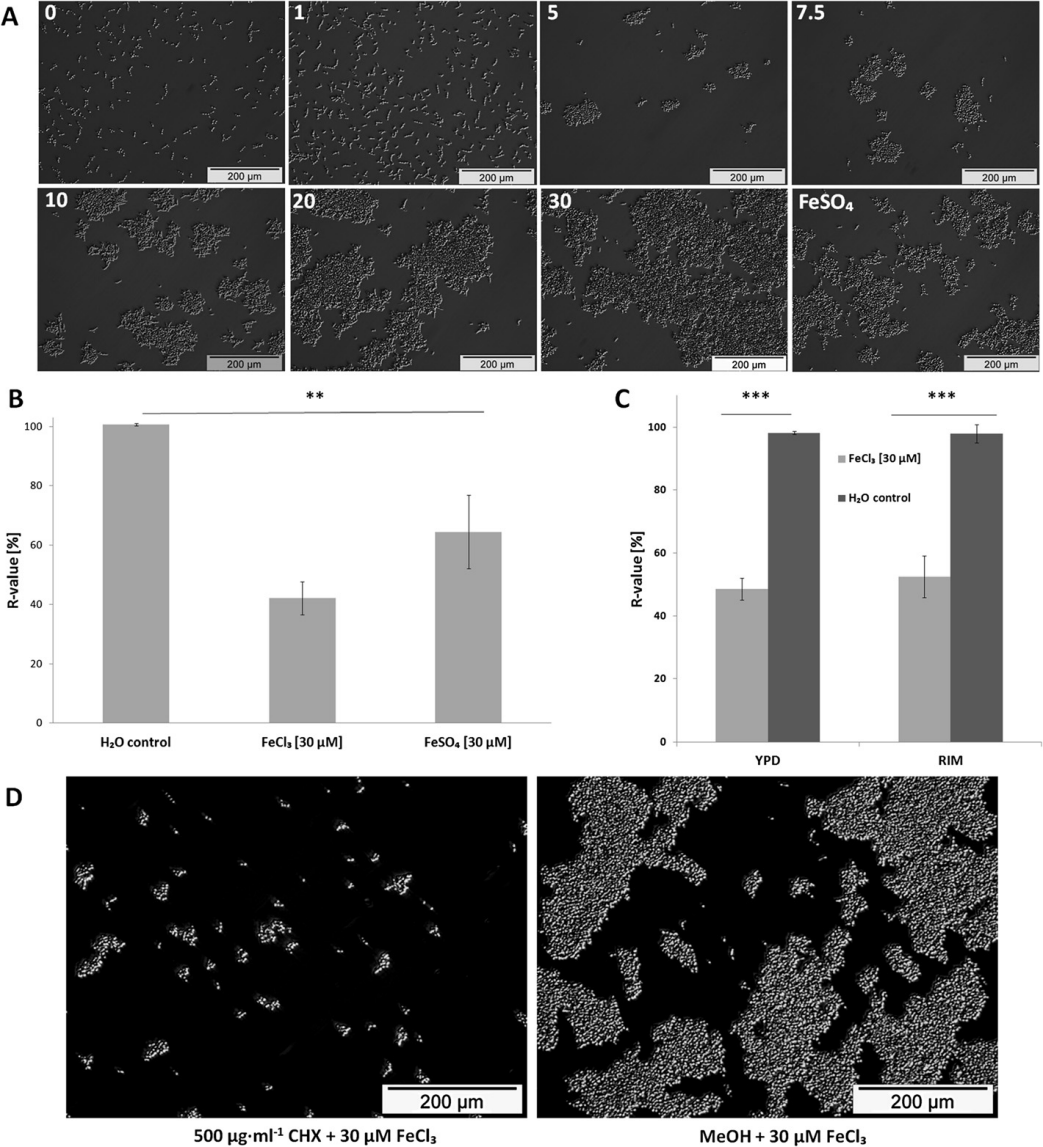
**Iron induced concentration dependent flocculation of *C. albicans *cells. **(**A**) Microscopic analysis. *C. albicans* SC5314 (WT) was incubated with different FeCl_3_ concentrations (indicated at the top left hand of each sub panel) or with 30 μM FeSO_4_ in RPMI at 30°C for 2 h. (**B**) Relative sedimentation rates of WT cells. Flocculation of cells was triggered by 30 μM FeCl_3_ or 30 μM FeSO_4_ in RPMI and sedimentation rates were determined after incubation at 30°C for 2 h. Means and standard deviations of three independent samples are shown (n = 3). ** denotes *P* < 0.01 (student’s *t*-test). (**C**) Relative sedimentation rates of WT cells pre-cultured in the sufficient iron (YPD) or restricted iron medium (RIM) at 30°C for 3 h. Flocculation of cells was triggered by 30 μM FeCl_3_ in RPMI and sedimentation rates were determined after incubation at 30°C for 2 h. Means and standard deviations of three independent samples are shown (n = 3). *** denotes *P* < 0.001 (student’s *t*-test). (**D**) Microscopic analysis of cycloheximide (CHX) or MeOH pre-treated cells. *C. albicans* SC5314 was pre-treated either with 500 μg ml^-1^ CHX or MeOH in RPMI at 30°C for 15 min. Iron or water were subsequently added and cells were incubated at 30°C for 2 h.

During cultivation of *C. albicans* SC5314 wild type (WT) in RPMI containing different FeCl_3_ concentrations (0, 1, 5, 7.5, 10, 20 and 30 μM) at 30°C, we observed flocculation of cells in an iron concentration dependent manner (Figure 1A). Flocs of cells could be seen at 5 μM and visibly increased from 7.5 to 30 μM Fe^3+^. Flocculation was induced when 30 μM FeSO_4_ were used as sole iron source instead of FeCl_3_. However, flocculation in response to FeSO_4_ was less pronounced at that iron concentration compared to 30 μM FeCl_3_ as quantified by measuring sedimentation rates (Figure 1B) as previously described [33].

Flocculation was also induced in yeast nitrogen base (YNB) medium containing 30 μM FeCl_3_ compared to 1.2 μM basal Fe^3+^ concentration (information given by the manufacturer), thus showing that the induction of flocculation was independent from the medium used (see Additional file 1).

Cells may possess internal iron stores from pre-cultivation in an iron sufficient medium. Thus, we investigated whether the iron content of the medium used during pre-cultivations influenced the dependence of the flocculent phenotype on the iron concentration in RPMI.

*C. albicans* was either pre-cultivated in a medium with sufficient iron, i.e. the rich yeast extract-peptone-dextrose (YPD) medium, or starved for iron by pre-cultivation in a medium with restricted iron availability (restricted iron medium: RIM). RIM resulted from addition of the iron chelator bathophenanthroline disulfonate (BPS) to YPD medium. As shown in Figure 1C, flocculation due to exposure to 30 μM Fe^3+^ was independent on the pre-cultivation medium: WT cells starved for iron by pre-cultivation in RIM flocculated upon exposure to 30 μM Fe^3+^ with a similar sedimentation rate as cells pre-cultivated in YPD. During all later experiments, we pre-cultivated *C. albicans* in YPD and added 30 μM FeCl_3_ as iron source to the respective medium of the working culture unless it is mentioned otherwise.

Interactions between cells leading to flocculation occur via constituents of the cell wall, which favor physical (hydrophobic or electrostatic) or specific biochemical interactions. The cell wall of *C. albicans* comprises proteins which are frequently mannosylated and attached to the backbone of the cell wall formed by glucans and chitin [34]. To obtain further information about the flocculent phenotype, protein biosynthesis was inhibited by cycloheximide (CHX) 15 min prior to iron addition. A reduction in flocculation was observed after iron addition compared to an equally treated methanol control (Figure 1D). Thus, protein synthesis seemed to be required for induction of iron dependent flocculation.

### High extracellular iron levels led to accumulation of intracellular ROS

**Figure 2:**
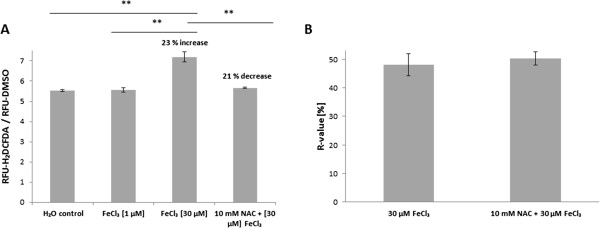
**High extracellular iron concentrations increased intracellular ROS levels.** (**A**) Determination of intracellular ROS production. WT cells were exposed to 0 (H_2_O control), 1 or 30 μM FeCl_3_ in RPMI at 30°C for 10 min. Additionally, cells were exposed to 30 μM FeCl_3_ together with 10 mM NAC. Means and standard deviations are shown from one representative experiment where all samples were derived from the same pre-culture. ** denotes *P* ≤ 0.01 (student’s *t*-test). All experiments were repeated 2 – 4 times from independent pre-cultures with similar results. (**B**) Influence of ROS on flocculation. Flocculation of cells was triggered by 30 μM FeCl_3_ in RPMI with or without 10 mM NAC. After 2 h incubation at 30°C, sedimentation rates were determined as described in the experimental part. Means and standard deviations of three independent samples are shown (n = 3).

Iron is a potent inducer of reactive oxygen species (ROS) under aerobic conditions. Ferric iron is reduced to ferrous iron by superoxide formed as byproduct of respiration. The resulting ferrous iron is oxidized by hydrogen peroxide to the extremely reactive hydroxyl radical. Thus, uptake of iron leads to the accumulation of toxic ROS and, correspondingly, accumulation of ROS can be used as indicator of iron uptake, if all other conditions are kept constant. ROS levels were determined using 2,7′-dichlorodihydrofluorescein diacetate (H_2_DCFDA) which is a cell permeable, oxidant sensitive agent widely used for intracellular ROS determination [35-38]. Compared to a water control, exposure of cells to 30 μM (high) but not to 1 μM (low) iron led to an increase in ROS generation by 15 - 40%. This effect could be reversed by the ROS scavenger N-acetyl cysteine (NAC), when added to the cells together with iron (Figure 2A).

Flocculation is frequently induced in yeasts as a response to stress [33,39]. As we had observed that high iron levels (30 μM) induced both flocculation as well as ROS accumulation while 1 μM Fe^3+^ did not, we investigated whether a relationship exists between the flocculation phenotype and iron induced oxidative stress. We determined the sedimentation rates of cells exposed to 30 μM iron and of cells exposed to the same iron concentration together with NAC. However, NAC did not prevent iron induced flocculation and the same sedimentation rates were obtained from both samples (Figure 2B). Thus, iron induced flocculation and ROS accumulation were not related to each other.

### MCFO expression was induced by low iron levels

**Figure 3:**
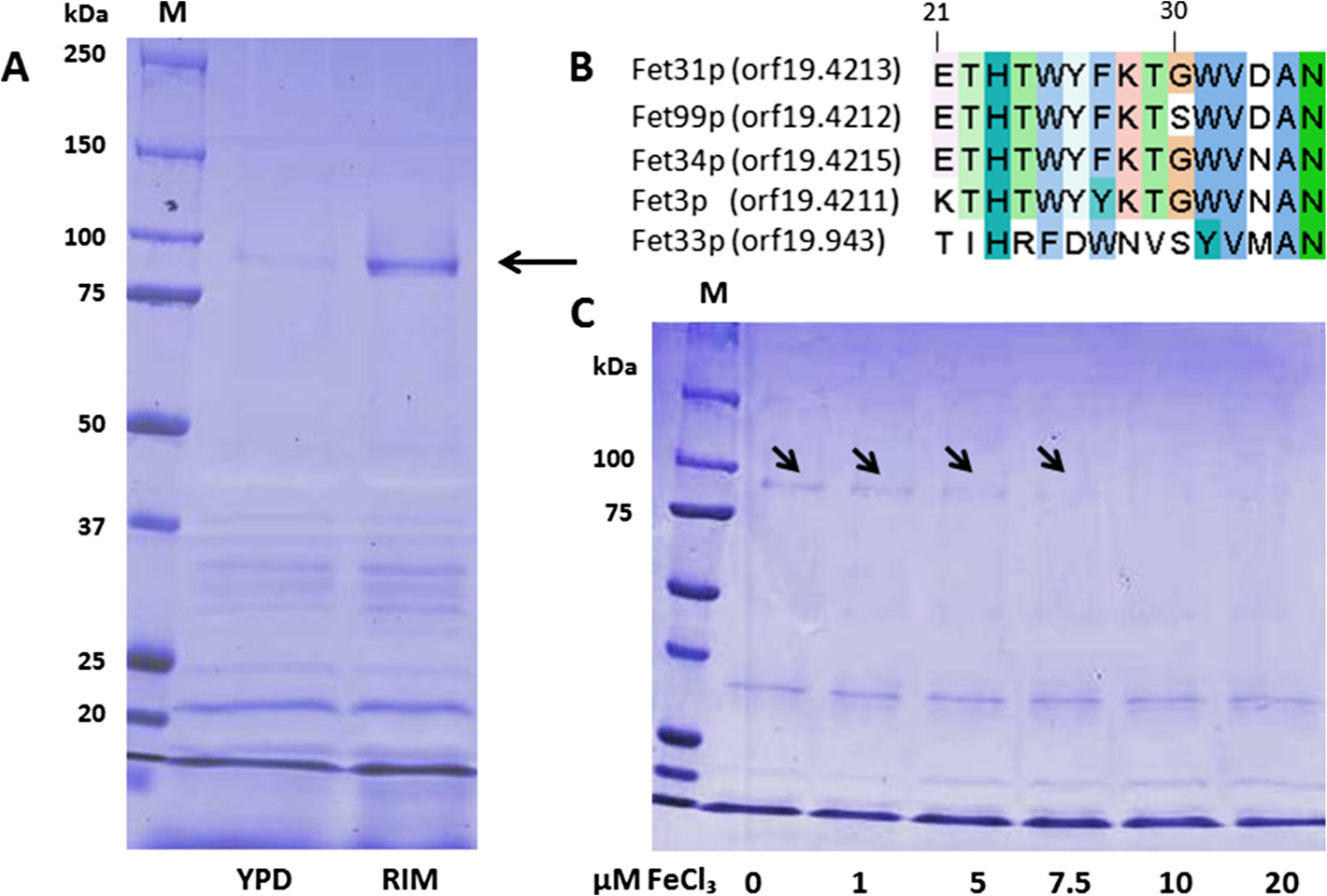
**MCFOs expression was regulated by iron levels.** (**A**) SDS-PAGE analysis of proteins extracted by heating whole yeast cells of *C. albicans* SC5314. Cells were cultivated in sufficient iron (YPD) or restricted iron (RIM) medium at 30°C for 5 h, and proteins were extracted as described in the experimental part. (**B**) Multiple sequence alignment (MSA) of the first 15 amino acids (aa) (given in the single letter code) after excision of a predicted 20 aa signaling peptide of MCFOs. The alignment was performed using CLUSTALW2 and displayed with the Jalview editor (http://www.ebi.ac.uk/Tools/msa/clustalw2/). The selected proteins are: Fet3p [UniProtKB: Q59NF9], Fet31p [UniProtKB: Q59NF7], Fet33 [UniProtKB: Q5A503], Fet34p [UniProtKB: Q59NF5] and Fet99p [UniProtKB: Q59NF8]. (**C**) SDS-PAGE analysis of MCFOs, which were extracted from cells grown in RPMI supplemented with different iron concentrations at 30°C for 3 h.

The expression of genes involved in iron uptake is regulated by iron availability. HAIU genes are induced under restricted iron conditions and repressed under high iron concentrations [23]. As mentioned above, members of the corresponding protein families are present in the plasma membrane of *C. albicans*. Heating whole microbial cells resuspended in phosphate buffers to elevated temperatures was already described as a method for the extraction of proteins associated with the cell wall or with the plasma membrane of different microorganisms [40-42]. We applied a similar approach by briefly boiling *C. albicans* cells grown in YPD medium or RIM. Proteins involved in HAIU were expected to be more abundant in cells cultivated in RIM compared to YPD. Extracted proteins were separated by SDS PAGE and visualized by coomassie staining. A protein band (80–100 kDa), which was significantly accumulated in RIM (Figure 3A), was analyzed by MALDI-TOF MS, MS/MS and N-terminal Edman degradation for identification. N-terminal sequencing of the protein extracted from the respective gel band resulted in the identification of the amino acid sequence KTHTxYYKTGxVNAN (amino acids given in the single letter code) which corresponds to the N-terminal sequence of the MCFO Fet3p (KTHTWYYKTGWVNAN) after cleavage of a predicted 20 amino acid signal peptide (Figure 3B). In the genome of *C. albicans*, five MCFO encoding genes are present. These are *FET3* (orf19.4211), *FET31* (orf19.4213), *FET33* (orf19.943), *FET34* (orf19.4215) and *FET99* (orf19.4212). The K21 residue is unique for Fet3p among *C. albicans* MCFOs (Figure 3B). Additionally, a glutamic acid peak appeared at residue 21, but was less intense than the lysine peak. This is indicative for the MCFOs Fet31p, Fet34p and Fet99p (Figure 3B). MALDI-TOF MS-analysis led to the identification of three peptide peaks specific for Fet34p and two peaks specific for Fet3p in addition to one peak shared between Fet34p and Fet3p, another peak shared between Fet3p, Fet31p and one peak shared between Fet3p, Fet31p and Fet99p (Table 1)*.* MS-MS analysis of the peak appearing at 1384.7 m/z unequivocally confirmed the presence of Fet34p in the excised band. Taken together, these data indicated the presence of at least Fet3p and Fet34p in the protein extract. However, presence of Fet31p and Fet99p is also possible and could neither be confirmed nor excluded. In general, all *C. albicans* MCFOs apart from Fet33p, are highly conserved among each other as Fet31p, Fet34p and Fet99p have an amino acid sequence identity ranging between 75 – 83% compared to Fet3p [15].

**Table 1:** Peptide peaks obtained from MS-MALDI-TOF analysis of the MCFOs band

**Peptide peaks [m/z]**	**MCFO**
998.5	Fet3p
1384.7	Fet34p
1389.7	Fet3p
1399.7	Fet34p
1507.8	Fet3p, Fet31p
1726.9	Fet3p, Fet34p
1838.9	Fet34p
1867.0	Fet3p, Fet31p, Fet99p

Previous gene expression experiments in *C. albicans* had reported that *FET34* expression was regulated by iron availability, as expression of this gene was induced under restricted iron compared to sufficient iron conditions [23,43]*.* Thus, we further investigated the dependence of MCFOs expression on iron concentrations in the growth medium. According to information given by the supplier, RPMI medium does not contain iron salts and can be considered as medium with very low basal iron levels. Thus, the concentrations of FeCl_3_ added to this medium were taken as total Fe^3+^ concentration. Increasing ferric iron concentrations led to significant decreases of MCFOs levels as determined by SDS PAGE and subsequent coomassie staining of proteins (Figure 3C). When iron concentrations equaled or exceeded 7.5 μM, hardly any protein band was visible. Taken together, these results confirm that the expression levels of extracted MCFOs were dependent on the iron ion concentration in the growth medium.

### Deletion of *HOG1* induced components of the HAIU pathway independent of iron availability

**Figure 4:**
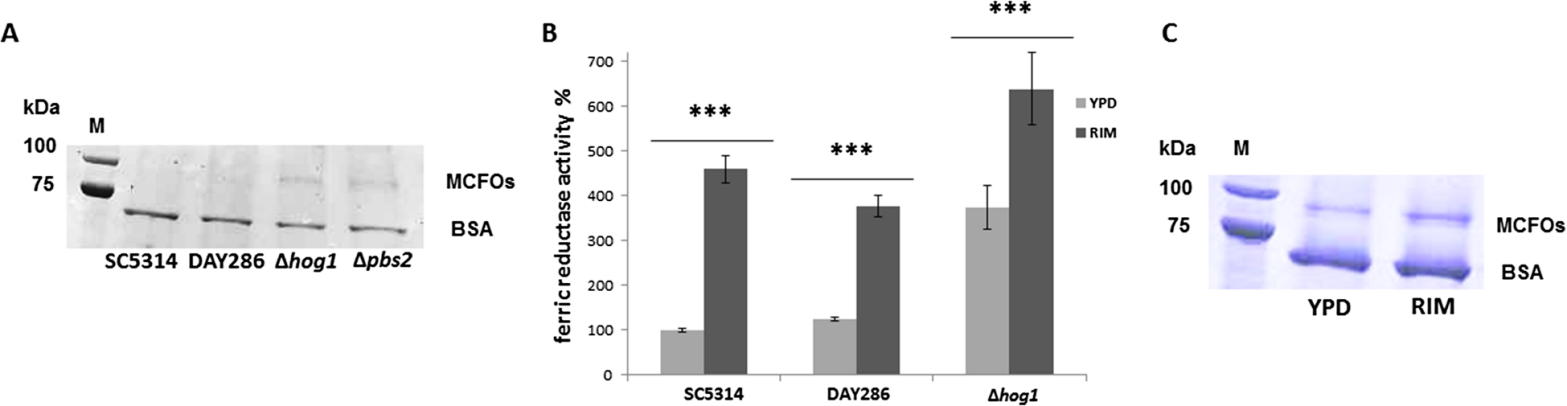
**Deletion of *HOG1 *led to de-repression of MCFOs and to increased ferric reductase activity.** (**A**) SDS-PAGE analysis of MCFOs extracted from the WT (SC5314), the reference strain (DAY286), Δ*hog1* (JMR114) and Δ*pbs2* (JJH31) mutants grown in YPD at 30°C for 16 h. For the whole gel see Additional file 2. (**B**) Cell surface ferric reductase activity of SC5314 (WT), DAY286 (reference strain) and Δ*hog1* (JMR114) under both restricted iron (RIM) and sufficient iron (YPD) conditions. Mean values and standard deviations of three independent experiments (n = 3) are shown. *** denotes *P* < 0.001 (student’s *t*-test). The ferric reductase of activity of the WT strain (SC5314) grown in YPD was set as 100%. (**C**) SDS-PAGE analysis of MCFOs extracted from Δ*hog1* (JMR114) grown in sufficient iron (YPD) or restricted iron (RIM) medium at 30°C for 3 h. Identity of the MCFOs was confirmed by mass spectrometry. For the whole gel see Additional file 3.

Previously, de-repression of genes involved in iron uptake (*FET34*, *FTR1*, *FRE10* and *RBT5*) was reported in the Δ*hog1* mutant by whole genome gene expression profiling of cells grown under sufficient iron conditions [27]. As the expression of these genes is usually repressed by sufficient iron conditions and only induced by restricted iron conditions [23] (for MCFOs see Figure 3), we investigated the function of Hog1p in the response of *C. albicans* to iron. We first confirmed elevated amounts of MCFO proteins in Δ*hog1* and Δ*pbs2* deletion mutants in comparison to the wild type (WT, SC5314) and the reference strain (DAY286) which was best seen in cells grown in YPD overnight (Figure 4A, see Additional file 2 for the complete gel). The identity of the MCFO proteins was proven by MS/MS analysis of the peptide at 1726.9 m/z (data not shown). Increased amounts of MCFOs were observed in two different, independently constructed Δ*hog1* and Δ*pbs2* mutants (see Table 2 for the strains used in this study [31,44]; data are shown for only one of the mutant strains). As proteins, which are usually used as gel loading controls, are cytosolic proteins and not present in the cell wall, we had added BSA to the extracted proteins to demonstrate that all lanes were loaded with the same total amount of protein. Fortunately, all bands in the gels showed an additional *C. albicans* protein band at molecular weights below 37 kDa, which had the same intensity in all samples so that it could be used as indicator of the amount of extracted protein (see Additional files 2 and 3 and also Figure 3). In RPMI the intensity of this band usually was slightly lower than the intensity of the MCFO band (MCFO : control = 1,1). After a cultivation time of 5h in YPD the MCFO band had an intensity of approximately 50% of this control band (see Figure 3).

**Table 2:** ***C. albicans *strains used in this work**

**Strain**	**Genotype**	**Reference**
SC5314 (MYA-2876)	Wild type (WT)	[65]
DAY286	*ura3∆ ::λimm434/ura3∆ ::λimm434, iro1/iro1, ARG4::URA3::arg4::hisG/arg4::hisG, his1::hisG/his1::hisG*	[53]
JMR114 (Δ*hog1*)	*ura3∆ ::imm434/ura3∆ ::imm434, iro1/iro1, arg4::hisG/arg4::hisG,his1::hisG/his1::hisG, hog1::ARG4/hog1::URA3*	[54]
CNC13 (Δ*hog1*)	*ura3∆ *::*imm434/ura3∆ *::*imm434, iro1/iro1, his1∆ *::*hisG/his1∆ *::*hisG hog1*::*hisGURA3-* *hisG/hog1*::*hisG*	[44]
JJH31 (Δ*pbs2*)	*ura3∆ ::λimm434/ura3∆ ::λimm434, iro1/iro1, arg4::hisG/arg4::hisG,his1::hisG/his1::hisG, pbs2::ARG4/pbs2::URA3*	[54]
BRD3 (Δ*pbs2*)	*ura3∆ ::imm434/ura3∆ ::imm434, iro1/iro1, his1∆ ::hisG/his1∆ ::hisG pbs2∆ * : : cat/*pbs2∆ * :: cat-*URA3*-cat	[31]
hAHGI (Δ*hog1* + *HOG1)*	*CNC13*, *ACT1p-HOG1-GFP : : leu2/LEU2*	[31]

As *FRE10*, the major ferric reductase of *C. albicans*[45], was also reported to be de-repressed in the Δ*hog1* mutant (see above) [27], we determined cell surface ferric reductase activity of whole yeast cells using a previously published protocol [45]. As shown in Figure 4B, ferric reductase activities increased when the wild type (SC5314) and the reference strain (DAY286) were cultivated in RIM compared to YPD. This further highlights the induction of this class of proteins by low iron levels. Moreover, cell surface ferric reductase activity was increased in Δ*hog1* mutants compared to both SC5314 and DAY286 when cultivated in YPD (data are shown for only one of the mutant strains), showing that de-repression of these enzymes in Δ*hog1* mutants led to higher enzyme activities. However, the response of HAIU components to low iron concentrations was not completely eliminated in the Δ*hog1* mutants, as we still observed induction of MCFOs expression (Figure 4C; see Additional file 3 for the complete gel) as well as increased ferric reductase activity when the Δ*hog1* mutant was cultivated in RIM (Figure 4B; data from only one of the mutants are shown). Thus deletion of *HOG1* led to both increased MCFOs expression as well as increased cell surface reductase activity, and both were further increased by iron restriction.

### *C. albicans* flocculation in response to high iron concentrations was dependent on both Hog1p and Pbs2p kinases

**Figure 5:**
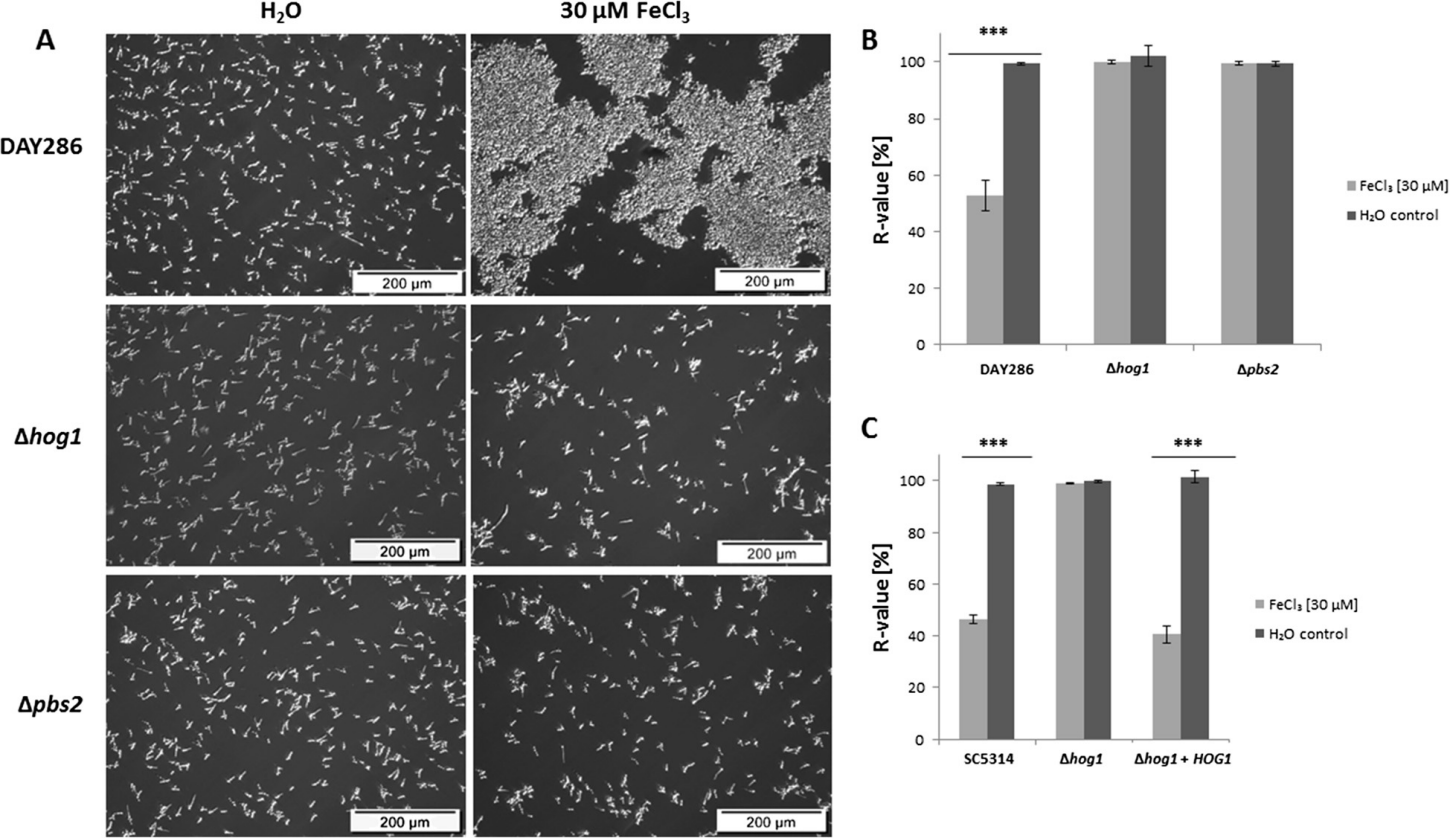
**High iron mediated flocculation was absent in Δ*hog1 *and Δ*pbs2 *mutants.** (**A**) Microscopic analysis of DAY286, Δ*hog1* (JMR114) and Δ*pbs2* (JJH31) upon exposure to iron. (**B**) Relative sedimentation rates of the reference strain (DAY286) and of Δ*hog1* (JMR114) and Δ*pbs2* (JJH31) mutants incubated in RPMI containing 30 μM FeCl_3_ or water (control) at 30°C for 2 h. Means and standard deviations of three independent samples are shown (n = 3). *** denotes *P* < 0.001 (student’s *t*-test). (**C**) Relative sedimentation rates of the WT (SC5314), Δ*hog1* (CNC13) and Δ*hog1* + *HOG1* (hAHGI) incubated in RPMI containing 30 μM FeCl_3_ or water (control) at 30°C for 2 h. The hAHGI strain carries the *HOG1* gene fused to *GFP* under control of the *ACT1* promoter and integrated in the *LEU2* locus [31]. Means and standard deviations of three independent samples are shown (n = 3). *** denotes *P* < 0.001 (student’s *t*-test).

We had observed that high iron concentrations induced a flocculent phenotype in WT cells (Figure 1). Thus, we investigated whether this phenotype was also dependent on the kinases Hog1p and Pbs2p. Interestingly, microscopic analysis and cell sedimentation assays showed that flocculation was absent in both Δ*hog1* and Δ*pbs2* mutants after exposure to high Fe^3+^, while still induced in the reference strain DAY286 (Figure 5A and B). When *HOG1* was re-integrated as fusion protein with GFP (strain hAHGI, Table 2), flocculation was restored after exposure to high iron concentrations as shown by measuring cell sedimentation rates (Figure 5C). Thus, the induction of flocculation was dependent on *HOG1* and *PBS2.* Moreover, we observed flocculation of Δ*hog1*, when 10% human plasma was added to the medium (data not shown). Thus, Δ*hog1* cells are generally still able to aggregate. Both observations indicate that Hog1p is specifically required for this iron-induced flocculent phenotype. The requirement of protein synthesis for flocculation was confirmed for the reference strain DAY286 (see Additional file 4A and B).

To ensure that iron was taken up by Δ*hog1* and Δ*pbs2* cells, we determined Fe^3+^ levels in culture supernatants of the reference strain DAY286 and the deletion mutants Δ*hog1* and Δ*pbs2* after an incubation time of 15 min. All three strains removed iron with the same efficiency from the growth medium (Table 3). Moreover, we observed increased intracellular ROS generation in Δ*hog1* cells after incubation with 30 μM FeCl_3_ (see Additional file 5), indicating intracellular activity of iron and thus iron uptake by those cells. In agreement with previous reports [36], we observed higher basal ROS production in Δ*hog1* cells compared to DAY286 cells.

**Table 3:** **Fe**^**3+ **^**removal from growth medium by *C. albicans *strains**

**Strain**	**Iron content of supernatant after 15 min at 30°C [% of starting conditions]**
DAY286	1.8 ± 0.8
Δ*hog1*	1.3 ± 0.47
Δ*pbs2*	2.6 ± 0.2

Starting Fe^3+^ concentrations of 30 μM were set as 100%.

### Hog1p was activated by high iron concentrations

**Figure 6:**
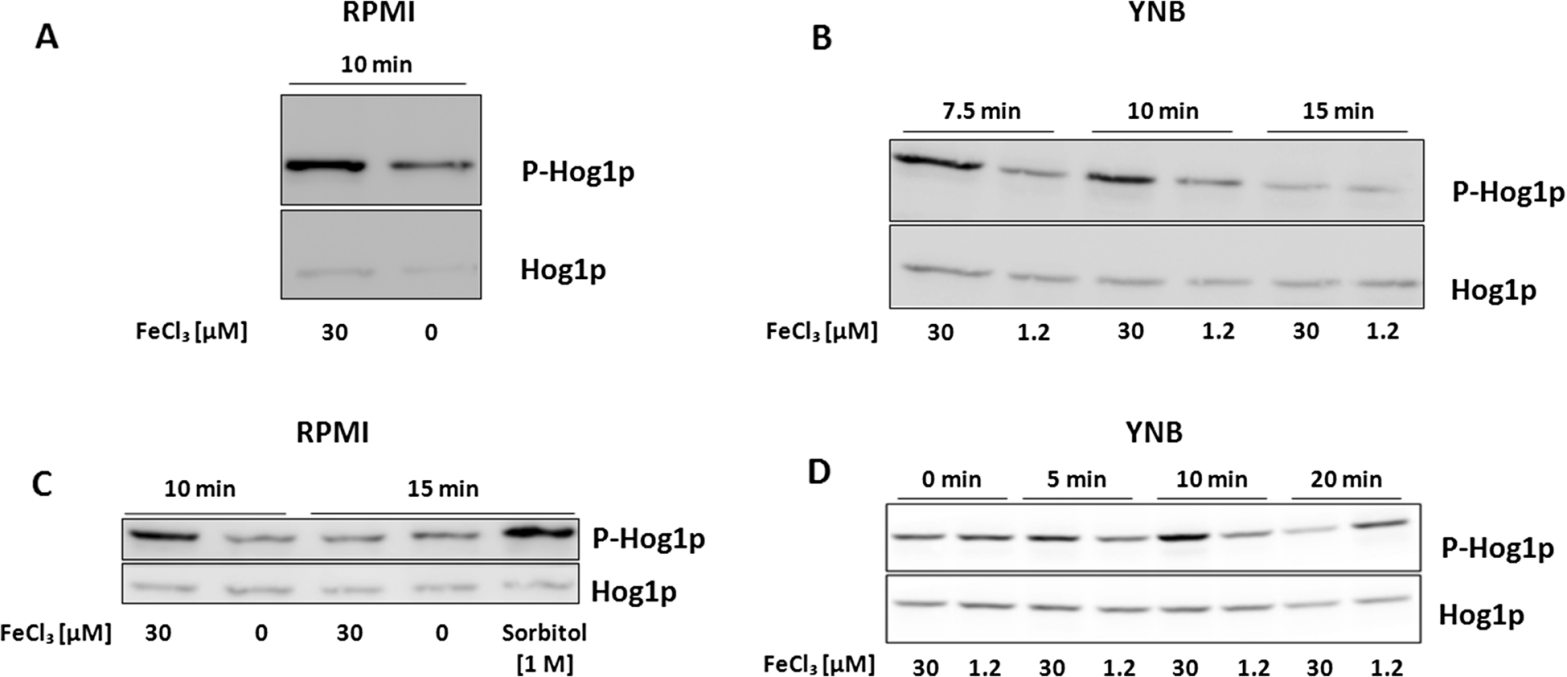
**The HOG pathway was activated by exposure to high iron levels.** (**A**) Western blot analysis of phosphorylated Hog1p (P-Hog1p) in *C. albicans* SC5314 (WT) cells exposed to 0 or 30 μM FeCl_3_ in RPMI at 30°C for 10 min. 5 μg total protein per sample were separated by SDS-PAGE. Phosphorylated Hog1p was detected by exposure of the membrane for 100 sec (for P-Hog1p) and 130 seconds (for Hog1p) after HRP reaction. (**B**) Western blot analysis of phosphorylated Hog1p in *C. albicans* SC5314 cells exposed to 30 μM or 1.2 μM FeCl_3_ in YNB medium for 7.5, 10 or 15 min at 30°C. 16 μg total protein per sample were separated by SDS-PAGE. Phosphorylated Hog1p was detected by exposure of the membrane for 100 sec (for P-Hog1p) and 130 seconds (for Hog1p) after HRP reaction. (**C**) Western blot analysis of phosphorylated Hog1p (P-Hog1p) in *C. albicans* DAY286 cells exposed to 0 or 30 μM FeCl_3_ in RPMI at 30°C for 10 or 15 min. Sorbitol [1 M] was used as positive control. 12 μg total protein per sample were separated by SDS-PAGE. Phosphorylated Hog1p was detected by exposure of the membrane for 80 sec (for P-Hog1p) and 40 seconds (for Hog1p) after HRP reaction. (**D**) Western blot analysis of phosphorylated Hog1p in *C. albicans* DAY286 cells exposed to 30 μM or 1.2 μM FeCl_3_ in YNB medium for 0, 5, 10 or 20 min at 30°C. Procedures were the same as indicated above except the following: 16 μg protein per sample were loaded on the gel and the membrane was exposed for 20 sec (P-Hog1p) and 30 sec (Hog1p) respectively. The pictures were slightly rotated to obtain almost straight bands.

As loss of *HOG1* influenced the response of *C. albicans* to elevated iron concentrations we determined the phosphorylation (i.e. activation) state of Hog1p after exposure to high Fe^3+^ concentrations. As shown in Figure 6A, we observed significant hyper-phosphorylation of Hog1p when the wild type strain SC5314 was exposed to 30 μM Fe^3+^. However, Hog1p hyper-phosphorylation was only transient, as maximum phosphorylation was obtained only from 7.5 - 10 min after exposure to high Fe^3+^ (Figure 6B). Results were similar, when the reference strain DAY286 was used (Figure 6C, D). Hog1p phosphorylation was almost as strong after exposure to high Fe^3+^ concentrations as after exposure to sorbitol (positive control) (Figure 6C). But Hog1p was dephosphorylated already 15 min after the exposure to iron (Figure 6D).

### Hog1p was required for maintenance of *C. albicans* viability under high iron conditions

Since Hog1p appeared to be involved in the response of *C. albicans* to high iron concentrations, we investigated whether Hog1p could have any protecting effect on *C. albicans* against deleterious effects of exposure to high iron levels. Thus, we determined the viability of cells after exposure to 30 μM Fe^3+^ using the AlamarBlue® assay, which is an indicator of the metabolic activity of cells [46]. This fluorescence assay has been widely used to determine viability of different yeasts including *C. albicans*[47-49]. We observed that basal fluorescence signals were always higher for Δ*hog1* cells than for the reference strain DAY286 (data not shown). This could be due to the intrinsically enhanced mitochondrial activity of *HOG1* deficient cells [36].

Cells were exposed to 30 μM FeCl_3_ in RPMI and incubated at 30°C for 60 min. A decrease of the reduction rate of AlamarBlue®, i.e. of the viability, was observed for all tested strains. However, exposure to high iron levels led to a higher decrease of the signals obtained from the Δ*hog1* mutant (residual viability 46 ± 3%) compared to the reference strain (DAY286) (residual viability 81 ± 9.5%) and the wild type (SC5314) (residual viability 85%). These data indicate that the Δ*hog1* mutant was less resistant to high iron levels than the WT cells. However, after 2 days no apparent growth defects were observed when the strains SC5314 (WT), DAY286 (reference strain), Δ*hog1* and Δ*pbs2* were grown on RPMI agar supplemented with 30 μM FeCl_3_ compared to cells grown on the same medium containing 0 or 1 μM FeCl_3_, respectively (see Additional file 6). This would indicate that the reduced metabolic activity of the Δ*hog1* mutant under high iron conditions did not affect growth of *C. albicans* on the long term.

The lower reduction rate of AlamarBlue® after exposure of Δ*hog1* to high Fe^3+^ concentrations was probably not due to the more oxidized intracellular environment after exposure of Δ*hog1* cells to high iron concentrations, as Δ*hog1* cells had a higher basal ROS level than WT cells, but the basal AlamarBlue® signals were also higher. Thus, the intracellular oxidation state (indicated by the ROS level) did not directly correlate with AlamarBlue® signals.

## Discussion

Previous studies on Δ*hog1* mutants from *C. albicans* and *Cryptococcus neoformans* showed that deletion of *HOG1* led to the de-repression of several genes known to be upregulated under restricted iron conditions [27,50]. In *C. albicans*, this group of genes included *RBT5*, *FRE10*, *FTR1*, *FET34*, orf19.251, *PMH7*, *ECM331*, *CAT1*, *DDR48*, *YOR009* and *HSP12*[22,23,27].

Whether this phenotype was due to a direct involvement of Hog1p in the regulation of the iron responsive network or due to indirect effects, such as perturbations of copper metabolism, which may have impaired the functionality of iron uptake proteins was not yet studied.

As expected, high levels of extracellular iron increased the formation of intracellular ROS. Thus, we used intracellular ROS levels together with the removal of iron from growth medium as indicators of iron entry into the cells. We detected increased basal ROS levels in the Δ*hog1* mutants, as previously reported [36]. These ROS levels were further increased by exposure to 30 μM Fe^3+^ confirming that iron was taken up by Δ*hog1* cells. Moreover, iron ions were removed from the growth medium with the same efficiency by Δ*hog1* as by the reference (DAY286) cells. Thus, Hog1p dependent phenotypes of the *C. albicans* response to iron were not due to iron uptake deficiencies, but could be rather due to the involvement of Hog1p in the response to iron availability. This is supported by our data on the transient hyper-phosphorylation of Hog1p during exposure of cells to high iron concentrations.

Elevated iron concentrations induced a flocculent phenotype of *C. albicans*, which was dependent on the presence of both Hog1p and Pbs2p, as well as on protein synthesis. As high iron concentrations led to increased phosphorylation of Hog1p, this could induce the synthesis of proteins of which some mediate cell aggregation. This iron triggered activation of Hog1p is likely not related to oxidative stress, as the potent radical scavenger NAC did not prevent the flocculent phenotype upon exposure to high iron concentrations, while it decreased intracellular ROS levels. For the closely related yeast *S. cerevisiae*, a function of *Sc*Hog1p in cell aggregation was reported, in that hyperactive *Sc*Hog1p mutants resulted in increased flocculation [51].

First hints on an involvement of Hog1p in the response of *C. albicans* to iron came from the observation of the de-repression of several iron uptake genes in the Δ*hog1* mutant under otherwise repressive conditions [27]. In agreement with these gene expression data, we observed increased MCFOs protein levels and ferric reductase activity in Δ*hog1* mutants. Furthermore we found that MCFOs were also de-repressed in Δ*pbs2* mutants, indicating that the *HOG1* mediated regulation of MCFOs was dependent on *PBS2*. Remarkably, induction of these components in RIM was not strictly dependent on Hog1p, as this induction was also observed in the Δ*hog1* mutant. Thus deletion of *HOG1* de-repressed components of the iron uptake system, and this elevated basal level was further enhanced when iron availability was limited.

Hog1p was shown to be essential for *C. albicans* under oxidative stress conditions [30]. Our data indicated that the absence of *HOG1* reduced the metabolic activity of the cells after exposure to high iron concentrations compared to wild type cells. Taking in account that exposure of Δ*hog1* cells to high iron concentrations further increased the comparably high basal intracellular ROS levels in the mutant, the decreased viability of the Δ*hog1* mutant under such conditions could be due to elevated oxidative stress. However, other mechanisms independent from Hog1p were also described for the initiation of oxidative stress responses [52]. These mechanisms could allow also the mutant strains to adapt to the stress conditions so that the reduced viability was observed only as immediate response and did not lead to significant growth defects.

It has yet to be elucidated which elements downstream of Hog1p provide the link between the HOG pathway and factors which regulate reductive iron uptake. As many Hog1p repressed genes, including those involved in iron uptake (*FET34*, *FRE10*, *FTR1* and *RBT5*), were also found to be repressed by Tup1p [27], a role for this global co-repressor downstream of Hog1p could be assumed. Indeed, a role of Tup1p in regulating iron uptake has been reported [17]. However, the details remain to be elucidated.

In this study, we used several single gene deletion mutants which were generated by different approaches [31,44,53,54]. All mutant strains were descendants of the strain CAI-4 [55], in which both copies of *IRO1* are deleted. Additionally, all strains ectopically express *URA3.*

*IRO1* is a gene that encodes a transcription factor with a potential role in iron utilization. Expression of *IRO1* in a Δ*aft1 S. cerevisiae* strain restored growth in iron depleted media. However, a role of *IRO1* in *C. albicans* iron metabolism is not confirmed [56]. On the other hand, ectopic expression of *URA3* has been shown to affect several features of *C. albicans*, such as hyphal morphology, adhesion, virulence and cellular proteome in addition to Ura3p activity [57,58].

In all of our experiments, the DAY286 reference strain behaved similar to the WT SC5314. Additionally, CNC13 and JMR114 (Δ*hog1*) as well as BRD3 and JJH31 (Δ*pbs2*) showed similar features. Thus, no effects of the ectopic expression of *URA3* or the absence of *IRO1* were observed.

## Conclusions

We report here for the first time in fungi, that the conserved stress activated MAP kinase Hog1p of *C. albicans* is involved in the response to changes in extracellular iron levels. Previous studies had only shown that deletion of *HOG1* led to the de-repression of HAIU components in this fungus under otherwise repressive conditions. We found that repression of HAIU components of the reductive pathway by Hog1p occurs independently of environmental iron availability. Exposure of *C. albicans* to high iron concentrations renders Hog1p hyper-phosphorylated. Thus, our results suggest that Hog1p has a dual role in *C. albicans* iron homeostasis. On the one hand basal Hog1p activity permanently reduces expression of HAIU components and on the other hand hyper-activity of Hog1p leads to the activation of a specific response towards high iron concentrations.

## Methods

### Strains, media and culture conditions

*C. albicans* strains used in this study are listed in Table 2. DAY286, JMR114 and JJH31 were purchased from the Fungal Genetics Stock Centre (Kansas, USA) [59]. Strains CNC13, BRD3 and hAHGI were kind gifts from Jesús Plá and co-workers (Madrid, Spain) [31,44].

Routinely, all strains were cultivated overnight (16 – 24 h) from frozen glycerol stocks in 20 or 50 ml YPD medium (Sigma-Aldrich Y1375) at 30°C. Growth was followed by measurements of optical densities (OD) of cultures at λ = 600 nm (OD_600_) in transparent 96 well plates by the μQuant microtiter plate reader (Biotek, Bad Friedrichshall, Germany) in triplicates (each 180 μl).

Cells from overnight cultures were diluted to an OD_600_ ~ 0.2 in YPD medium or restricted iron medium (RIM) and grown until early exponential phase (3 h) at 30°C (pre-culture). RIM was produced by adding 200 μM of the potent iron chelator bathophenanthroline disulfonate (BPS) to YPD (Table 4). Cells were harvested from the pre-culture by centrifugation at 4500 x *g* and room temperature (RT) for 5 min, followed by resuspension in the respective growth medium. Growth media used in this study are summarized in Table 4. RPMI1640 is a medium comprising no iron salts, YNB is a defined medium with a basal concentration of 1.2 μM Fe^3+^ (information from the suppliers). All liquid media used in this study were prepared in ultrapure Milli-Q (MQ) water (Millipore, Billerica, USA) and sterilized by filtration using 0.2 μm bottle top filters (Milian). During all experiments, ferric chloride (FeCl_3_, Sigma-Aldrich) was chosen as ferric iron source, while ferrous sulfate (FeSO_4_, Sigma-Aldrich) served as source for ferrous iron. All iron containing stock solutions were freshly prepared immediately before use. For cultivations exceeding a cultivation time of 10 min in iron supplemented media, iron stock solutions were sterile filtered by 0.2 μm Minisart sterile filters (Sartorius, Göttingen, Germany) before being added to the media.

**Table 4:** Growth media used in this work

**Medium**	**Composition**
RPMI	8.4 g L^-1^ RPMI 1640 (Sigma-Aldrich R1383), 2 g L^-1^ glucose, 0.165 M 3-(N-morpholino propanesulfonic acid (MOPS), adjusted to pH 7.3 with 10 N NaOH
YNB	6.7 g L^-1^ Yeast Nitrogene Base (Sigma Y1250), 2 g L^-1^ glucose, 0.165 M 3-(N-morpholino propanesulfonic acid (MOPS), adjusted to pH 7.3 with 10 N NaOH
YPD	Sufficient iron medium: Yeast extract (10 g L^-1^) peptone (20 g L^-1^) dextrose (20 g L^-1^) (Sigma-Aldrich Y1375)
RIM	Restricted iron medium; YPD + 200 μM bathophenantroline disulfunate (BPS) (Sigma 146617)

### Protein analysis

For the extraction of MCFOs, an overnight culture was diluted in YPD to an OD_600_ ~ 0.2 and grown until the early exponential phase (pre-culture). Working cultures were prepared by resuspending *C. albicans* cells from the pre-culture in 20 ml of the respective medium at an OD_600_ ~ 0.3. Cultures were incubated at 30°C for 3 – 5 h or at an OD = 0.1 in 20 – 50 ml medium for 16–17 h (overnight cultivations). After incubation, cells were collected by centrifugation (4500 × *g*, 5 min, RT) and washed twice with PBS, pH 7.4 (8.0 g NaCl, 0.2 g KCl, 1.44 g Na_2_HPO_4_, 0.24 g KH_2_PO_4_). The supernatant was removed and the pelleted cells were washed with 1 ml PBS and subjected to a further short centrifugation step (4500 × *g*, 1 min, RT). The supernatant was removed and 30 – 100 μl PBS were added to the wet cell pellet. Proteins from resuspended cells were extracted by boiling at 90°C for 10 min. The suspension was centrifuged at 10000 × *g* and 4°C for 10 min and the supernatant was transferred to a new 1.5 ml Eppendorf tube. This centrifugation step was repeated once to remove residual cells. The protein extract (supernatant) was subjected to protein determination using bicinchoninic acid [60]. Equal protein concentrations in all samples were obtained by diluting the samples with PBS according to the concentration of the least concentrated sample. All protein samples were mixed with 5x protein sample buffer (1.5 g sodium dodecyl sulphate (SDS), 1.116 g dithiothreitol, 0.015 g bromphenol blue, 7.5 ml 0.5 M Tris HCl pH 6.8, 7.5 ml glycerol) in a ratio of 4:1, boiled at 95°C for 10 min and stored at −20°C until use. Proteins (60 – 70 μg) were separated on freshly prepared 1 D SDS-gels containing 12.5% running gel and 4% stacking gel (Rotiphorese® Gel 30 (37.5:1), Roth, Karlsruhe, Germany). Gels were run at 120 V for up to 3 h (unless otherwise mentioned), before staining with coomassie staining solution (0.25% Coomassie-G25, 50% H_2_O, 42% Ethanol, 8% acetic acid) at RT for 30 min followed by destaining with distilled water (dH_2_O) overnight with an occasional interval in destaining solution (50% H_2_O, 42% Ethanol, 8% acetic acid) for no longer than 15 minutes. Gel documentation was performed with the GS-800 gel scanner (Bio-Rad, München, Germany). In the figures only those parts of the gels are shown, which contain the bands, which are relevant for the results described here. Occasionally, after documentation distorted bands were bent to obtain almost straight bands.

For MALDI-TOF peptide mass fingerprinting protein bands were cut out from 1D SDS-gels, reduced and carboxamidomethylated, and then subjected to in-gel tryptic digestion. The resulting peptides were extracted, desalted using ZipTip devices (Millipore, Bedford, USA) and analyzed by MALDI-TOF-MS using a Bruker Ultraflex time-of-flight mass spectrometer (Bruker Daltonics, Bremen, Germany). Laser induced dissociation of selected peptides for sequence confirmation was performed on the same instrument. Identification of proteins was performed with the mascot search engine at http://www.matrixscience.com/.

For N-terminal sequencing, proteins were blotted on polyvinylidene fluoride (PVDF) membranes and stained with Coomassie G-25 at room temperature for 5 min. Background color was removed by incubation in destaining solution for 30 min. Bands of interest were cut off from the membrane and subjected to N-terminal sequencing using a 494A HT Protein Sequencer (Applied Biosystems) [61].

To investigate Hog1p phosphorylation, an overnight culture was diluted to an OD_600_ ~ 0.2 in YPD and allowed to grow at 30°C for another 3 h. Then cells were resuspended in 20 ml of the respective medium at an OD_600_ ~ 0.3 or 0.1 and were incubated with or without addition of FeCl_3_ at 30°C for the given time points. Occasionally, cells were washed with the same medium before adding iron. As positive control for Hog1p phosphorylation, cells were incubated with 1 M of the osmotic stress inducer sorbitol in RPMI at 30°C for 15 min. Protein preparation and western blotting were performed as previously described [62] with some modifications. Briefly, cells were frozen in liquid nitrogen and disrupted with a Microdismembrator (Mikro-Dismembrator U, B. Braun Biotech International, Melsungen, Germany) and the resulting cell powder was resuspended in extraction buffer (10 mM sodium phosphate buffer, pH 8.5 containing 5 mM NaCl, 5 mM KCl, 11 g L^-1^ glucose, supplemented with 1x protease inhibitor (cOmplete, mini EDTA free) and 1 - 2x phosphatase inhibitor (PhosSTOP, Roche)). Protein content of each sample was determined as described above. Protein samples were separated in the same gels as indicated above. Gels were run at 80 V for 30 min and subsequently at 120 V for 90 min before proteins were blotted on PVDF membranes. Nonfat dried milkpowder (Euroclone, Italy) was used as blocking agent. Blots were probed with anti-phospho p38 MAPK (Thr180/Tyr182) 3D7 rabbit mAB (Cell Signaling Technology) and with horse-radish-peroxidase (HRP)-linked anti-rabbit IgG antibody (Cell Signaling Technology) to detect phosphorylated Hog1p. Bands were visualized by chemiluminescence using the ECL Advance Western Blotting Detection Kit (GE Healthcare). Membranes were stripped with Re-Blot stripping buffer (Millipore) and blots were probed with anti-Hog1p (y-215) sc 9079 rabbit polyclonal IgG (Santa Cruz Biotechnology) and the HRP-linked anti-rabbit antibody mentioned above to detect total Hog1p content.

### Flocculation and sedimentation assays

*C. albicans* cells from an overnight culture were diluted in YPD to an OD_600_ of 0.2 and allowed to grow to the early logarithmic phase. Cells were pelleted (4500 × *g*, 5min, RT) and resuspended in 2 ml of the respective medium containing different iron concentrations in 14 ml polypropylene (PP) round bottom falcon tubes (BD sciences, USA) at an OD_600_ of 0.1. Flocculation was observed microscopically after incubating cells at 30°C for up to 2 h. Alternatively, 20 ml cultures were prepared in 100 ml shaking flasks. Flocculation was quantified by determination of relative sedimentation rates (R-values) of cells based on a previously published protocol [33]. Briefly, 1 ml of the cell suspension was transferred to a plastic cuvette after incubation at 30°C for 2 h. OD_600_ was determined directly after vortexing the cell suspension (OD1) and after additional 15 min without vortexing (OD2). The R-value was calculated as percentage of OD2 relatively to OD1 (OD2/OD1 * 100) and reflects a decrease in OD with increased sedimentation rate. Each experiment contained three independent replicates, and the mean of the three obtained R-values was taken as a final result.

### Intracellular ROS determination

*C. albicans* cells from an overnight culture were diluted in YPD to an OD_600_ of 0.2 and allowed to grow to the early logarithmic phase. Cells were pelleted (4500 x *g*, 5min, RT), washed once with RPMI and resuspended in 2 ml RPMI with or without iron in round bottom falcon tubes at an OD_600_ of 0.1. Cells were incubated at 30°C for 10 min and immediately pelleted and washed twice with MQ-H_2_O. Cells from all samples were resuspended each in 1.2 ml water and each sample was split in two 600 μl samples containing either 70 μM CM-H_2_DCFDA (Invitrogen) or the same volume of DMSO. From those stocks, 3 x 180 μl were pipetted into the wells of a 96 well plate and incubated in the dark at 30°C for 30 min [36]. Fluorescence intensity was quantified by measuring relative fluorescence intensities (RFUs) using the Synergy 4 fluorescence microtiter plate reader (BioTek Instruments GmbH) at an excitation wavelength of 485 nm and an emission wavelength of 528 nm. ROS accumulation was calculated with respect to background fluorescence of the sample: ROS accumulation = (RFU-H_2_DCFDA/RFU-DMSO). To reverse ROS accumulation, the radical scavenger N-acetyl cysteine (Sigma-Aldrich) was used at 10 mM final concentration together with iron.

### Determination of iron levels in growth media and culture supernatants

Ferric iron concentrations in media and culture supernatants were indirectly determined by reducing total ferric iron to ferrous iron by ascorbic acid at low pH and measuring ferrous iron content through the chromogenic iron chelator bathophenanthroline disulfonate (BPS). Briefly, *C. albicans* cells were prepared as described in the flocculation part. Cells were incubated in 2 ml RPMI (OD_600_ ~ 0.1) containing 30 μM FeCl_3_ at 30°C for 15 min. A medium sample lacking iron was used as negative control, while medium supplemented with 30 μM FeCl_3_ without cells represented the starting conditions and was equally treated. After incubation, cells were removed by centrifugation (4500 x *g*, 5 min, RT), and 880 μl from the supernatants were mixed with 100 μl of 10 mM ascorbic acid and 20 μl of 50 mM BPS. All samples were acidified by addition of 10 μl 32% HCl and 180 μl of this mixture were pipetted in a transparent 96 well plate and the absorption of the BPS · Fe^2+^ complex was measured in triplicates at λ = 535 nm [63,64] immediately after acidification. Absorption of the iron free sample was used for background correction of all other samples. For each strain, three samples were measured. Each sample was obtained from an independent culture. The whole experiment was repeated three times.

### Determination of cell surface ferric reductase activity

*C. albicans* DAY286 and Δ*hog1* overnight cultures were diluted in YPD to an OD_600_ of 0.2 in RIM or YPD medium. All cultures were incubated at 30°C until early exponential phase. After this period of growth, ferric reductase assay was performed according to [45] with minor modifications. Briefly, early exponential cells were washed once with MQ-H_2_O (4500 x *g*, 5 min, RT), resuspended in assay buffer (50 mM sodium citrate, 5% glucose, pH 6.5) and shaken in round bottom falcon tubes at 30°C for 15 min. FeCl_3_ and BPS were then added at a final concentration of 1 mM each, to give a final volume of 2 ml. Cells were incubated at 30°C for additional 5 min, pelleted (8000 x *g*, 3 min, RT) and the OD_520_ of the supernatant was determined (3 x 180 μl) (λ = 520 nm). The results are shown as percentage of DAY286 ferric reductase activity in YPD. Each experiment was performed three times.

### Viability test

Viability of cells was measured using the AlamarBlue® assay (Invitrogen), which indicates particularly the metabolic activity of a culture. *C. albicans* cells were prepared as described in the flocculation part and resuspended in 2 ml RPMI with addition of 30 μM FeCl_3_ or MQ-H_2_O at an OD_600_ of 0.1. Cells were incubated at 30°C for 60 min and immediately pelleted and washed once with MQ-H_2_O. The cells were resuspended in 2 ml MQ-H_2_O and 3 x 162 μl from each sample was added to 3 × 18 μl AlamarBlue® which were previously pipetted in three wells of a 96 well plate. The fluorescence intensity was quantified (t = 0) with the Synergy 4 fluorescence microtiter plate reader (BioTek Instruments GmbH) at an excitation wavelength of 540 nm and an emission wavelength of 590 nm. The reagent was incubated at 30°C for 30 min and the fluorescence intensity was quantified again (t = 30 min). The difference to the values obtained at t = 0 was taken as indicator of the viability of the cells and the relative metabolic activity was calculated according to: Relative metabolic activity (%) = 100 × (RFU_iron_/RFU_MQ-H2O_). Experiments for reference strain (DAY286) and Δ*hog1* (JMR114) were performed three times (n = 3) in total and means of the three experiments were taken as final results. Experiment for the WT strain (SC5314) was performed once as a control.

## Abbreviations

HAIU: High affinity iron uptake; MCFO: Multicopper ferroxidase; RIM: Restricted iron medium; BPS: Bathophenanthroline disulfonate; PBS: Phosphate buffered saline; ROS: Reactive oxygen species; CHX: Cycloheximide; RT: Room temperature; OD: Optical density; RFU: Relative fluorescence unit; HRP: Horse radish peroxidase.

## Competing interests

The authors declare that they have no competing interests.

## Authors’ contributions

HEJK designed and performed all experiments, analyzed results and prepared figures and additional files. MN performed mass spectrometric analysis and wrote the respective procedures in the methods part. HEJK and MN analyzed mass spectrometric data. PPM contributed extensively to experimental design and result analysis. PPM edited a late version of the manuscript. UB supervised the whole project, designed experiments and analyzed results. HEJK and UB wrote the manuscript. All authors have read and approved the manuscript.

## Supplementary Material

Additional file 1 **Induction of *C. albicans* flocculation by 30 μM FeCl**_**3**_** in YNB Microscopic analysis of the reference strain (DAY286) after exposure to 30 μM or 1.2 μM FeCl**_**3**_** in YNB.** Cells were incubated at 30°C for 2 h. Click here for file

Additional file 2 **Deletion of *HOG1* led to de-repression of MCFOs.** Whole gel of the SDS-PAGE analysis shown in Figure. 4A. Δ*hog1* JMR114; Δ*pbs2* JJH31. Click here for file

Additional file 3 **SDS-PAGE analysis of proteins extracted from the Δ*hog1* mutant cultivated in YPD medium and RIM. Whole gel of the SDS-PAGE described in Figure **4**C.** Click here for file

Additional file 4 **Effect of cycloheximide pre-incubation on iron induced flocculation.** (A) Relative sedimentation rates of DAY286 cells treated with cycloheximide (CHX) *C. albicans* DAY286 was pre-treated either with 500 μg ml^-1^ CHX or MeOH in RPMI at 30°C for 15 min. Iron or water were subsequently added and cells were incubated at 30°C for 2 h. Sedimentation rates were determined as described in the experimental part. Means and standard deviations of three independent samples are shown (n = 3). ** denotes P ≤ 0.01 (student’s *t*-test). (B) Microscopic analysis of CHX or MeOH pre-treated cells (see A). Click here for file

Additional file 5 **ROS determination in the Δ*hog1* (JMR114) mutant.** Experiments for ROS accumulation in Δ*hog1* cells were performed twice (n = 2). Means and standard deviations are shown of one representative experiment where all samples were derived from the same pre-culture. *** denotes *P* < 0.001 (student’s *t*-test). Click here for file

Additional file 6 **Deletion of *HOG1* had no influence on *C. albicans *growth in media with high iron concentrations.** The WT (SC5314), the reference strain (DAY286), and the Δ*hog1* (JMR114) and Δ*pbs2* (JJH31) mutants were diluted in YPD each to ca. 0.5 · 10^6^ cells ml^-1^ and further diluted in 1:10 steps. 5 μl of each cell suspension were dropped on RPMI agar plates containing 0 (RPMI), 1 or 30 μM FeCl_3_. Plates were incubated for 2 d at 30°C before pictures were taken. All plates were prepared in triplicates and one representative for each plate is shown. Click here for file
