# Supplementary figures and images for: Involvement of the mitogen activated protein kinase Hog1p in the response of Candida albicans to iron availability
Source: BMC Microbiol. 2013 Jan 24;13:16. doi: 10.1186/1471-2180-13-16 (PMC3637358; doi:10.1186/1471-2180-13-16)

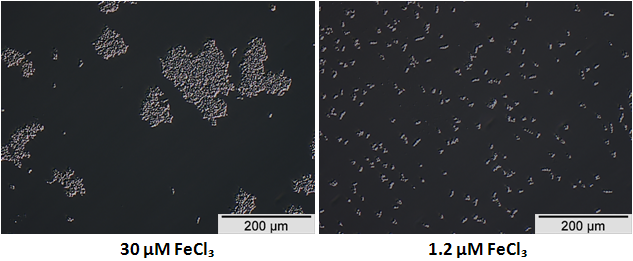

Supplement: Additional file 1 — Induction of C. albicans flocculation by 30 μM FeCl3 in YNB Microscopic analysis of the reference strain (DAY286) after exposure to 30 μM or 1.2 μM FeCl3 in YNB. Cells were incubated at 30°C for 2 h. [file 1471-2180-13-16-S1.tiff]

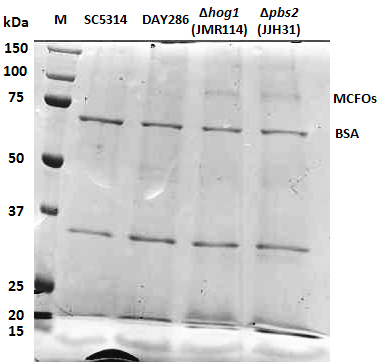

Supplement: Additional file 2 — Deletion of HOG1 led to de-repression of MCFOs. Whole gel of the SDS-PAGE analysis shown in Figure. 4A. Δhog1 JMR114; Δpbs2 JJH31. [file 1471-2180-13-16-S2.tiff]

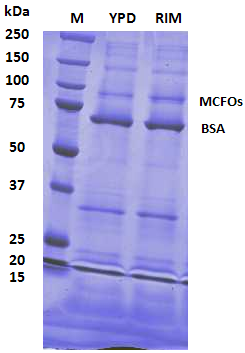

Supplement: Additional file 3 — SDS-PAGE analysis of proteins extracted from the Δhog1 mutant cultivated in YPD medium and RIM. Whole gel of the SDS-PAGE described in Figure 4C. [file 1471-2180-13-16-S3.tiff]

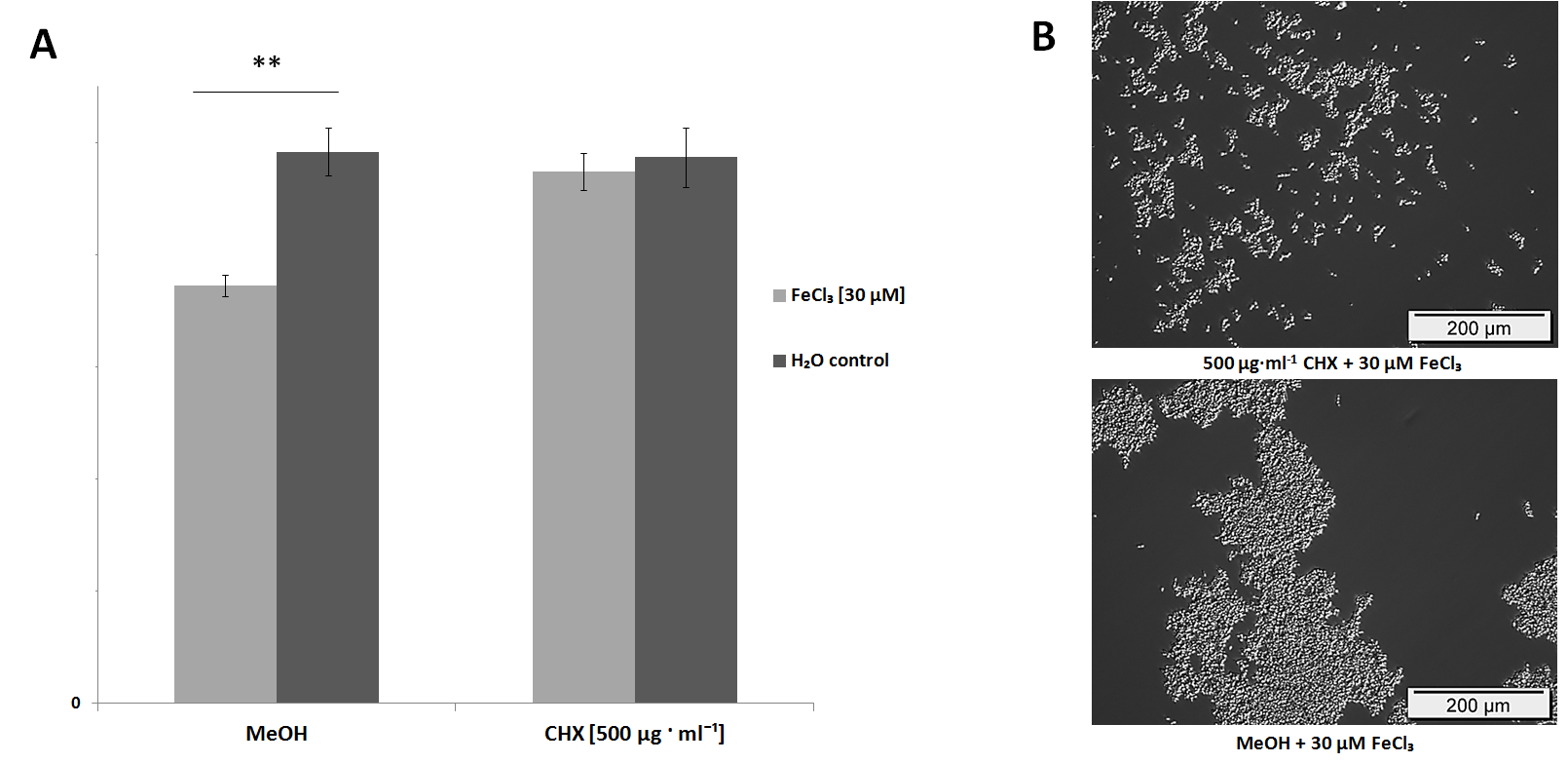

Supplement: Additional file 4 — Effect of cycloheximide pre-incubation on iron induced flocculation. (A) Relative sedimentation rates of DAY286 cells treated with cycloheximide (CHX) C. albicans DAY286 was pre-treated either with 500 μg ml-1 CHX or MeOH in RPMI at 30°C for 15 min. Iron or water were subsequently added and cells were incubated at 30°C for 2 h. Sedimentation rates were determined as described in the experimental part. Means and standard deviations of three independent samples are shown (n = 3). ** denotes P ≤ 0.01 (student’s t-test). (B) Microscopic analysis of CHX or MeOH pre-treated cells (see A). [file 1471-2180-13-16-S4.tiff]

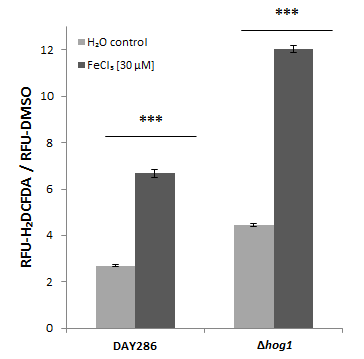

Supplement: Additional file 5 — ROS determination in the Δhog1 (JMR114) mutant. Experiments for ROS accumulation in Δhog1 cells were performed twice (n = 2). Means and standard deviations are shown of one representative experiment where all samples were derived from the same pre-culture. *** denotes P < 0.001 (student’s t-test). [file 1471-2180-13-16-S5.tiff]

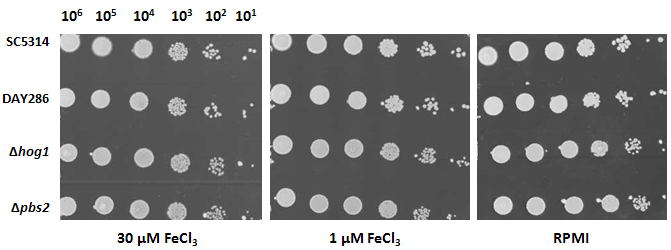

Supplement: Additional file 6 — Deletion of HOG1 had no influence on C. albicans growth in media with high iron concentrations. The WT (SC5314), the reference strain (DAY286), and the Δhog1 (JMR114) and Δpbs2 (JJH31) mutants were diluted in YPD each to ca. 0.5 · 106 cells ml-1 and further diluted in 1:10 steps. 5 μl of each cell suspension were dropped on RPMI agar plates containing 0 (RPMI), 1 or 30 μM FeCl3. Plates were incubated for 2 d at 30°C before pictures were taken. All plates were prepared in triplicates and one representative for each plate is shown. [file 1471-2180-13-16-S6.tiff]
